# Differentiation Between Women With Vulvovaginal Symptoms Who are Positive or Negative for *Candida* Species by Culture

**DOI:** 10.1155/S1064744901000369

**Published:** 2001

**Authors:** Iara M. Linhares, Steven S. Witkin, Shirlei D. Miranda, Angela M. Fonseca, Jose A. Pinotti, William J. Ledger

**Affiliations:** 1 Department of Gynecology Hospital das Clinicas University of Sao Paulo Sao Paulo Brazil; 2 Division of Immunology and Infectious Diseases Department of Obstetrics and Gynecology Weill Medical College of Cornell University 515 East 71st Street New York NY 10021 USA

## Abstract

*Objective:* To investigate whether clinical criteria could differentiate between women with vulvovaginitis who were culture positive or negative for vaginal * Candida* species.

*Methods:* Vulvovaginal specimens were obtained from 501 women with a vaginal discharge and/or pruritis. Clinical information and wet mount microscopy findings were obtained. All specimens were sent to a central laboratory for species identification.

*Results:* A positive culture for *Candida* species was obtained from 364 (72.7%) of the specimens. *C. albicans* was identified in 86.4% of the positive cultures, followed by *C. glabrata* in 4.5%, *C. parapsilosis* in 3.9%, *C. tropicalis* in 2.7% and other *Candida * species in 1.4%.Women with a positive *Candida* culture had an increased utilization of oral contraceptives (26.1% vs. 16.8%, *p* = 0.02) and antibiotics (8.2% vs. 0.7%, *p* = 0.001), and were more likely to be pregnant (9.1% vs. 3.6%, *p* = 0.04) than the culture-negative women. Dyspareunia was more frequent in women without *Candida* (38.0% vs. 28.3%, *p* = 0.03) while vaginal erythema (*p* =  0.01) was more common in women with a positive *Candida* culture.

*Conclusions:* Although quantitative differences were observed, the presence of vaginal *Candida* vulvovaginitis cannot be definitively identified by clinical criteria.


Infect Dis Obstet Gynecol 2001;9:221–225

### Differentiation between women with vulvovaginal symptoms who are positive or negative for *Candida* species by culture

**Iara M. Linhares[1,2], Steven S. Witkin[2], Shirlei D. Miranda[1], Angela M. Fonseca[1], Jose A. Pinotti[1] and William J. Ledger[2]**

[1]*Department of Gynecology, Hospital das Clinicas, University of Sao Paulo, Sao Paulo, Brazil*
[2]*Division of Immunology and Infectious Diseases, Department of Obstetrics and Gynecology, Weill Medical College of Cornell University, New York, NY*

***Objective:*** To investigate whether clinical criteria could differentiate between women with vulvovaginitis who were culture positive or negative for vaginal *Candida* species.
***Methods:*** Vulvovaginal specimens were obtained from 501 women with a vaginal discharge and/or pruritis. Clinical information and wet mount microscopy findings were obtained. All specimens were sent to a central laboratory for species identification.
***Results:*** A positive culture for *Candida* species was obtained from 364 (72.7%) of the specimens. *C. albicans* was identified in 86.4% of the positive cultures, followed by *C. glabrata* in 4.5%, *C. parapsilosis* in 3.9%, *C. tropicalis* in 2.7% and other *Candida* species in 1.4%. Women with a positive *Candida* culture had an increased utilization of oral contraceptives (26.1% vs. 16.8%, $p = 0.02$) and antibiotics (8.2% vs. 0.7%, $p = 0.001$), and were more likely to be pregnant (9.1% vs. 3.6%, $p = 0.04$) than the culture-negative women. Dyspareunia was more frequent in women without *Candida* (38.0% vs. 28.3%, $p = 0.03$) while vaginal erythema ($p = 0.01$) was more common in women with a positive *Candida* culture.
***Conclusions:*** Although quantitative differences were observed, the presence of vaginal *Candida* vulvovaginitis cannot be definitively identified by clinical criteria.

Key words: VULVOVAGINITIS; *CANDIDA* SPECIES; DIFFERENTIAL DIAGNOSIS

It is difficult to obtain accurate information regarding the prevalence and incidence of vulvovaginitis associated with a *Candida* species infection. Although up to 75% of women will acknowledge having had a vaginal *Candida* infection during their lifetime[1], this diagnosis is suspect. The skyrocketing sales of over-the-counter medications for *Candida* vaginitis, at a rate many times that of the number of infected women, highlights the prevalent overdiagnosis of this disorder. Many women, and unfortunately also many clinicians, label any vaginal discharge, itching, pain or burning as a 'yeast' infection. In three studies, more than half of the women with a supposed vaginal yeast infection were misdiagnosed[2–4].

Conversely, in many women a true vaginal *Candida* infection may remain unrecognized. Detection of a vaginal *Candida* infection by microscopic examination of a vaginal specimen diluted in potassium hydroxide is relatively insensitive, especially for non-albicans species[3,5]. False-positive microscopic examinations are also possible[5] and are

Supported by Janssen-Cilag, Sao Paulo, Brazil
Correspondence to: Steven S. Witkin, Ph.D., Department of Obstetrics and Gynecology, Weill Medical College of Cornell University, 515 East 71st Street, New York, NY 10021, USA. Email: switkin@mail.med.cornell.edu probably more common than generally suspected. It is also possible to have *Candida* vulvovaginitis with a false-negative *Candida* culture. At least 3000 organisms/ml are necessary to obtain a positive culture[6].

In an attempt to more accurately characterize symptomatic women with a positive *Candida* species vaginal culture, and to differentiate them from women with vaginal symptoms due to other causes, a study was initiated in three cities in Brazil.

### MATERIALS AND METHODS

This study was approved by the Clinical and Ethical Committee of Hospital das Clinicas, University of Sao Paulo, and informed written consent was obtained from all subjects. The study population consisted of 501 consecutive reproductive age women complaining of a vaginal discharge and/or vulvovaginal pruritis, seen as private patients in the Brazilian cities of Sao Paulo, Rio Grande de Sul and Salvador. Exclusion criteria included the use of immunosuppressive medications, vaginal medications or oral antifungal agents within the last 30 days.

Clinical and demographic data were collected at each center by a single participating physician. Signs and symptoms upon physical examination were standardized as much as possible between the different sites by providing common diagnostic criteria for erythema, edema, discharge and dysuria. Definitions were similar to those utilized by Eckert and colleagues[7].

Specimens were obtained by scraping the vaginal walls with a cotton swab and immediately transferring the contents to a glass slide. A drop of saline was added and diagnosis of *Candida* was based on the observed presence of mycelium (branched hyphal elements) or blastospores (the unicellular yeast form). A second specimen was placed in transport medium and shipped to a central clinical laboratory. Specimens were cultured on Sabouraud agar containing chloramphenicol. *Candida* species were identified by the automated Amphotericin B (ATB) express method.

Comparisons between women with positive or negative *Candida* cultures for quantitative variables were analyzed by the Student's *t*-test for independent samples. The $\chi^2$ test was used to compare qualitative variables between both groups. Findings were considered significant at $p < 0.05$.

### RESULTS

*Candida* was detected by culture in 364 (72.7%) of the subjects. The distribution of individual *Candida* species can be seen in Table 1. *C. albicans* was identified in 86.4% of the positive cultures, followed by *C. glabrata* (4.5%), *C. parapsilosis* (3.9%) and *C. tropicalis* (2.7%). A presumed identification of *Candida* was made by microscopic examination in 87.1% of women with a positive culture and in 5.1% of those with a negative culture. *Trichomonas vaginalis* was present in 2.9% and 1.4% of women with a negative and positive *Candida* culture, respectively. Clue cells were observed in 16.8% of women with a negative culture and in 9.1% of women with a positive *Candida* culture ($p = 0.01$).

For all analyses, the patients were divided into two groups based on the presence or absence of a positive *Candida* culture. Demographics of women in both groups are shown in Table 2. A higher percentage of black women, but not of white women or those of other races, were present in the culture-negative group (16.8%) than in the *Candida* culture-positive group (8.3%) ($p = 0.01$).

The relationship between predisposing factors and a positive or negative *Candida* culture is detailed in Table 3. Pregnancy (9.1% vs. 3.6%, $p = 0.04$), oral contraceptive usage (26.1% vs. 16.8%, $p = 0.02$) and current antibiotic use (8.2% vs. 0.7%, $p = 0.001$) were each associated with detection of a positive *Candida* culture. Conversely, a positive HIV serology (7.3% vs. 3.3%,

**Table 1** *Candida* species identified by culture

| Candida *species* | *Percentage women positive* |
|---|---|
| *albicans* | 86.4 |
| *glabrata* | 4.5 |
| *parapsilosis* | 3.9 |
| *tropicalis* | 2.7 |
| *krusei* | 0.9 |
| *guilliermondii* | 0.6 |
| *famata* | 0.3 |
| *pulcherrima* | 0.3 |
| *susitanii* | 0.3 |

**Table 2** Demographics of women with vulvovaginitis and a positive or negative culture for *Candida*

| | Candida ($n$ = 364) | *No* Candida ($n$ = 137) |
|---|---|---|
| Race | | |
| White | 79.0% | 73.7% |
| Black | 8.3% | 16.8%* |
| Oriental | 5.0% | 1.5% |
| Other | 7.7% | 8.0% |
| Age (years) | 32.2 (10.1)† | 33.8 (10.8)† |
| Sexually active | 93.1% | 84.7% |
| > 1 Sexual partner | 7.5% | 3.1% |

*$p = 0.01$ vs. black women with *Candida*; †standard deviation

**Table 3** Predisposing factors associated with *Candida* species culture-positive and -negative vulvovaginitis

| | Candida | *No* Candida |
|---|---|---|
| Diabetes | 2.8% | 4.4% |
| Pregnancy | 9.1%* | 3.6% |
| Oral contraception | 26.1%** | 16.8% |
| IUD usage | 6.6% | 11.0% |
| Corticosteroid usage | 2.2% | 2.2% |
| Antibiotic usage | 8.2%*** | 0.7% |
| HIV seropositive | 3.3% | 7.3%† |
| Prior STD | 9.7% | 17.5%†† |

*$p = 0.04$, **$p = 0.02$, ***$p = 0.001$ vs. women without *Candida*; †$p = 0.05$, ††$p = 0.01$ vs. women with *Candida*

$p = 0.05$) and prior history of a sexually transmitted disease (17.5% vs. 9.7%, $p = 0.01$) were associated with a negative *Candida* culture. The HIV-seropositive women were at the earliest stages of their disease.

The relationship between patient-reported signs and symptoms and *Candida* culture findings is shown in Table 4. There was considerable overlap between the two groups in complaints of a vaginal discharge, vulvar pruritis and burning, dysuria and dyspareunia. Only dyspareunia was significantly different between the two groups: 38.0% in women with a negative culture vs. 28.3% in those with a positive *Candida* culture ($p = 0.03$).

Clinical findings in the patient groups are shown in Table 5. Although there was an overall high degree of similarity between subjects regardless of their *Candida* culture status, women with a positive culture had a higher prevalence of vaginal erythema ($p = 0.01$).

**Table 4** Signs and symptoms of women with vulvovaginitis positive and negative for *Candida* by culture

| | Candida | *No* Candida |
|---|---|---|
| Vulvar pruritis | 83.2% | 82.5% |
| Vulvar burning | 61.8% | 66.4% |
| Vaginal discharge | 85.7% | 87.6% |
| Dyspareunia | 28.3% | 38.0%* |
| Dysuria | 19.8% | 21.9% |
| > 1 previous episode | 44.5% | 53.3% |
| Length of symptoms (days) | $18.6 \pm 8.6$ | $15.0 \pm 12.0$ |

*$p = 0.03$ vs. women with *Candida*

**Table 5** Clinical findings in women with vulvovaginitis and positive or negative for *Candida* by culture

| | Candida | *No* Candida |
|---|---|---|
| Vulvar edema | 10.7% | 9.5% |
| Vulvar erythema | 16.5% | 16.3% |
| Vaginal fissures | 18.4% | 16.1% |
| Vaginal erythema | 86.8%* | 59.2% |
| Leukorrhea | 84.9% | 88.6% |
| Excoriation | 9.9% | 8.8% |
| Cervical ectopy | 11.5% | 14.6% |
| Vesicles | 2.2% | 2.2% |
| Pustules | 2.2% | 2.2% |

*$p = 0.01$ vs. women without *Candida*

### DISCUSSION

Although some quantitative differences in the frequency of patient symptoms, clinical findings and predisposing factors were identified between groups of women who were culture-positive or culture-negative for *Candida* species, none of the evaluated criteria were pathognomonic for *Candida* in the vagina. Similar findings have been reported previously by others[2,6–8]. In a large study of women attending a sexually transmitted disease clinic, only 28% of 545 women with pruritis, burning or a vaginal discharge were *C. albicans*-culture positive[7]. Thus, in symptomatic women, a positive wet mount or culture for *Candida* is necessary to assess whether this organism is present in the vagina.

The findings in the present study of associations between a positive *Candida* culture in symptomatic women and current oral contraceptive and antibiotic usage and pregnancy parallel earlier reports[9–11]. We recognize that other studies have reported that women using oral contraceptives containing estrogen levels of 35 μg or less did not have an increased rate of candida vulvovaginitis[7,11,12]. This does not seem to be true, however, for our study population. The oral contraceptives used by the patients examined contained estrogen levels below 35 μg.

The etiology of the vaginal symptoms in the majority of our 137 *Candida* culture-negative patients remains undetermined. Bacterial vaginosis was a possible cause in 23 of these women, based on detection of clue cells by microscopy. An additional 4 women were positive for *T. vaginalis* by microscopic examination. Other possible causes of the observed symptoms, not evaluated in the present study, include allergic vaginitis, papillomavirus infection, desquamative vaginitis, cervicitis or estrogen deficiency. In addition, we acknowledge that in the *Candida* positive group, clinical signs and symptoms in at least some of the women may have been due to causes other than the presence of *Candida* species. However, the strong association between culture and wet mount suggests the presence of a high *Candida* concentration in the majority of the positive women and, therefore, an increased likelihood of *Candida*-related symptomatology.

Host factors must also be taken into consideration when attempting to diagnose vulvovaginitis. It has been reported that there is no association between the *Candida* concentration in the vagina and clinical symptoms[13]. Women with *Candida* counts as low as 100 organisms/ml may be highly symptomatic while some women with vaginal *Candida* concentrations as high as 10 000/ml may be asymptomatic. Thus, women who are allergic to *Candida* antigens or products[14] can become symptomatic even when vaginal concentrations of this organism are below the level detectable by culture. Furthermore, a low level of *Candida* in the vagina can synergize with histamine released in response to other allergens to induce prostaglandin $E_2$ production[15]. This, in turn, inhibits the cell-mediated immune response necessary to prevent *Candida* proliferation.

It is apparent that patient symptomatology, clinical examination and medical history are insufficient to distinguish vulvovaginitis associated with the presence of a *Candida* species from vulvovaginitis due to other causes. Initiation of antifungal treatment based solely on these criteria will be ineffective for the many women who are negative for this microorganism. In symptomatic women, a positive wet mount or culture for *Candida* species is necessary to determine whether this microbe is present.

### ACKNOWLEDGMENT

We thank Luiz Carlos Severo for the *Candida* species identification.

### REFERENCES

1. Hurley R, DeLouvois J. Candida vaginitis. *Postgrad Med J* 1979;55:645–7
2. Berg AO, Heidrich FE, Fihn SD, *et al*. Establishing the cause of symptoms in women in a family practice. *J Am Med Assoc* 1984;251:620–5
3. Nyirjesy P, Seeney SM, Grody MH, *et al*. Chronic fungal vaginitis: the value of cultures. *Am J Obstet Gynecol* 1995;173:820–3
4. Ledger WJ, Polaneczky MM, Yih MC, *et al*. Difficulties in the diagnosis of *Candida* vaginitis. *Infect Dis Clin Pract* 1999;9:66–9
5. Odds FC. Genital candidosis. *Clin Exp Dermatol* 1982;7:345–54
6. Schaaf VK, Perex-Stable EJ, Borchardt K. The limited value of symptoms and signs in the diagnosis of vaginal infections. *Arch Intern Med* 1990;150:1566–9
7. Eckert LO, Hawes SE, Stevens CE, *et al*. Vulvovaginal candidiasis: clinical manifestations, risk factors, management algorithm. *Obstet Gynecol* 1998;92:757–65
8. Sobel JD, Faro S, Force RW, *et al*. Vulvovaginal candidiasis: epidemiologic, diagnostic, and therapeutic considerations. *Am J Obstet Gynecol* 1998; 178:203–11
9. Spinillo A, Capuzzo F, Nicola S, *et al*. The impact of oral contraception on vulvovaginal candidiasis. *Contraception* 1995;51:293–7
10. Morton RS, Rashid S. Candidal vaginitis: natural history, predisposing factors and prevention. *Proc R Soc Med* 1977;70(Suppl 4):3–6

11. Ryley JF. Pathogenicity of *Candida albicans* with particular reference to the vagina. *J Med Vet Mycology* 1986;24:5–22
12. Gough PM, Warnock DW, Turner A, *et al*. Candidosis of the genital tract in non-pregnant women. *Eur J Obstet Gynecol Reprod Biol* 1985; 19:237–46
13. Meech RJ, Smith JM, Chew T. Pathogenic mechanisms in recurrent genital candidosis in women. *N Z Med J* 1985;771:1–5
14. Witkin SS, Jeremias J, Ledger WJ. A localized vaginal allergic response in women with recurrent vaginitis. *J Allergy Clin Immunol* 1988;81:412–16
15. Witkin SS, Kalo-Klein A, Galland L, *et al*. Effect of *Candida albicans* plus histamine on prostaglandin E2 production by peripheral blood mononuclear cells from healthy women and women with recurrent candidal vaginitis. *J Infect Dis* 1991;164:396–9

RECEIVED 03/29/01; ACCEPTED 07/18/01
